# Inverse relationship between chitobiase and transglycosylation activities of chitinase-D from *Serratia proteamaculans* revealed by mutational and biophysical analyses

**DOI:** 10.1038/srep15657

**Published:** 2015-10-23

**Authors:** Jogi Madhuprakash, Kishore Babu Bobbili, Bruno M. Moerschbacher, Tej Pal Singh, Musti J. Swamy, Appa Rao Podile

**Affiliations:** 1 Department of Plant Sciences, School of Life Sciences, University of Hyderabad, Gachibowli, Hyderabad, India; 2 School of Chemistry, University of Hyderabad, Gachibowli, Hyderabad, India; 3 Institute for Biology and Biotechnology of Plants, WWU, Münster University, Münster, Germany; 4 Department of Biophysics, All India Institute of Medical Sciences, New Delhi, India

## Abstract

*Serratia proteamaculans* chitinase-D (*Sp*ChiD) has a unique combination of hydrolytic and transglycosylation (TG) activities. The TG activity of *Sp*ChiD can be used for large-scale production of chito-oligosaccharides (CHOS). The multiple activities (hydrolytic and/or chitobiase activities and TG) of *Sp*ChiD appear to be strongly influenced by the substrate-binding cleft. Here, we report the unique property of *Sp*ChiD substrate-binding cleft, wherein, the residues Tyr28, Val35 and Thr36 control chitobiase activity and the residues Trp160 and Trp290 are crucial for TG activity. Mutants with reduced (V35G and T36G/F) or no (*Sp*ChiDΔ30–42 and Y28A) chitobiase activity produced higher amounts of the quantifiable even-chain TG product with degree of polymerization (DP)-6, indicating that the chitobiase and TG activities are inversely related. In addition to its unprecedented catalytic properties, unlike other chitinases, the single modular *Sp*ChiD showed dual unfolding transitions. Ligand-induced thermal stability studies with the catalytically inactive mutant of *Sp*ChiD (E153A) showed that the transition temperature increased upon binding of CHOS with DP2–6. Isothermal titration calorimetry experiments revealed the exceptionally high binding affinities for E153A to CHOS with DP2–6. These observations strongly support that the architecture of *Sp*ChiD substrate-binding cleft adopted to control chitobiase and TG activities, in addition to usual chitinase-mediated hydrolysis.

Chitin is one of the most abundant natural polysaccharides, and comprises (1 → 4) linked units of 2-acetamido-2-deoxy-β-D-glucopyranose residues (GlcNAc, **A**-unit) in a linear form. In contrast, chitosan is a soluble co-polymer made up of GlcNAc and 2-amino-2-deoxy-β-D-glucopyranose residues (GlcN, **D**-unit)^1^. The degradation products of chitin and chitosan are chito-oligosaccharides (CHOS), which have many potential biological applications particularly in the fields of food, medicine and agriculture^1^,^2^,^3^,^4^,^5^,^6^. However, CHOS are difficult to produce with well-defined structures. Most biological activities require CHOS with a degree of polymerization (DP) ≥ 4, and several enzymatic methods are available to generate CHOS with a DP ≥ 6 using specific chitinases^7^,^8^. Catalysis by glycoside hydrolase family 18 (GH18) chitinases proceeds *via* a substrate-assisted mechanism, which involves an oxazolinium ion intermediate^9^,^10^,^11^ and has a strong preference for a GlcNAc in the –1 subsite. If the fully/partially deacetylated CHOS bind such that GlcN is placed in the –1 subsite, they act as inhibitors of GH18 chitinases^12^,^13^.

Certain chitinases display transglycosylation (TG) activity as well as hydrolysis, forming new glycosidic bonds between donor and acceptor saccharides^14^,^15^,^16^ that can be used for the production of longer-chain and/or well-defined mixtures of CHOS with new or improved biological activities^17^. A single-domain GH18 chitinase-D from *Serratia proteamaculans* (*Sp*ChiD) possesses multiple functions, including chitobiase activity to release GlcNAc from CHOS and colloidal chitin, and a hyper-TG activity with CHOS (DP3–6) substrates, generating long-chain CHOS^14^. Although mutations have been used to improve the TG activity of *Sp*ChiD^18^,^19^, the overall efficiency of TG was inevitably limited by the enzyme-catalyzed hydrolysis of the product. Hence, there is a need to curtail the hydrolytic activity of *Sp*ChiD to improve the yield of TG products, which requires a careful understanding of the role of key residues at the substrate-binding cleft.

The *Sp*ChiD substrate-binding cleft contains four tryptophan residues (Trp114, Trp160, Trp290 and Trp395) with Trp160 and Trp290 flanking the entrance and Trp114 and Trp395 situated in the catalytic groove of the binding pocket (Fig. 1A). Half of the space in the *Sp*ChiD substrate-binding cleft is occupied by a loop (Asn30–Asp42), and the front face is equipped with Val35 and Thr36 (Fig. 1A), which may provide favorable chemical interactions with the incoming substrate^20^. The spatial alignment of these residues within the proximity of the substrate-binding cleft (PDB: 4LGX) may facilitate ligand binding and control the interplay between hydrolysis and TG activities. Therefore, we used fluorescence quenching studies in the presence or absence of CHOS to investigate the involvement of Trp residues in ligand binding, and determined the binding affinities using isothermal titration calorimetry (ITC). We also analyzed the ligand-induced thermal stability of *Sp*ChiD using differential scanning calorimetry (DSC).

The *Sp*ChiD crystal structure (PDB: 4LGX) combined with DSC and ITC data prompted us to test additional mutations to determine the role of the loop (Asn30–Asp42), and the Trp residues in the binding pocket, in TG activity. Phylogenetic analysis of 131 bacterial family-18 chitinases revealed that all the *Sp*ChiD-like single-domain chitinases from the Enterobacteriaceae clustered in a distinct group. The novel loop and tryptophan residues were highly conserved in 18 of these enzymes, confirming their divergence from other groups (Fig. 1B,C).

## Results

### Thermal stability and binding properties of *Sp*ChiD and the mutant E153A

**Figure 2:**
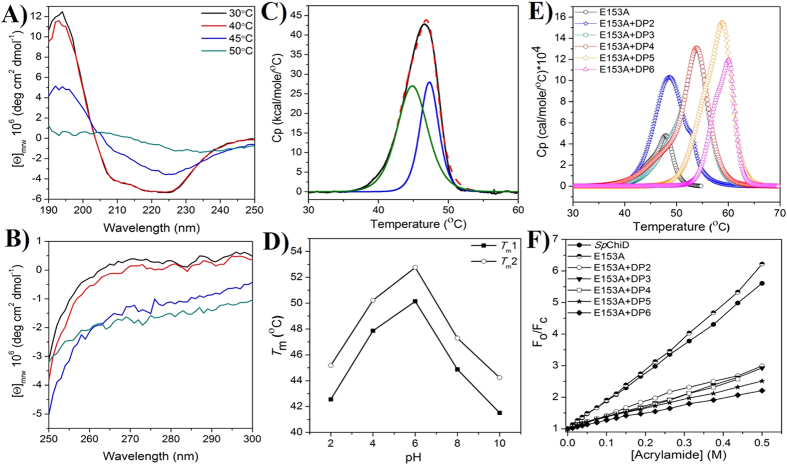
Spectroscopic and calorimetric analysis of SpChiD and mutant E153A. (**A**) Far and (**B**) near UV-CD spectra were recorded at different temperatures. (**C**) DSC thermogram of *Sp*ChiD alone after buffer baseline correction in 20 mM sodium phosphate, pH 8.0. Dotted line in red indicates deconvolution of the thermogram showing the presence of two distinct unfolding transitions. (**D**) DSC scans for *Sp*ChiD as a function of pH show that the protein is thermally stable at pH 6.0 and undergoes two distinct transitions centered at 50.1 °C and 52.7 °C. Values for the calorimetric enthalpy (Δ*H*c), Van’t Hoff enthalpy (Δ*H*v) and their ratio (Δ*H*c/Δ*H*v) corresponding to these transitions at different pH values are listed in Supplementary Table 1. (**E**) The effect of ligand binding on thermal stability of E153A was studied by pre-incubating the protein with ligands DP2–6, which represent the ligands (GlcNAc)_2–6_ unless otherwise specified. (**F**) The fluorescence emission data recorded in the absence and presence of ligands (DP2–6) was fitted to the linear curve fitting model, and the slope was considered as the Stern-Volmer constant (*K*_SV_). Stern-Volmer plot of *Sp*ChiD in the absence of ligands is also compared with the mutant E153A.

The far and near UV-circular dichroism (CD) spectra for *Sp*ChiD at 45 °C showed low signal intensities compared to the spectra recorded up to 40 °C, indicating perturbations in the structural elements. At 50 °C, we observed a collapse in the secondary and tertiary structures (Fig. 2A,B). The results suggested that the enzyme *Sp*ChiD alone was stable up to 40 °C. Increase in temperature by a factor of 5 °C resulted in changes in the final conformation of the protein. Pre-incubation of *Sp*ChiD for 20 min at different temperatures caused the activity at 45 °C to decline and a complete loss of activity at 50 °C, confirming the conclusions drawn from the CD spectral data (Supplementary Fig. 1).

Further, DSC studies showed that *Sp*ChiD exhibits two unfolding transitions (Fig. 2C) and is most stable at pH 6.0 (Fig. 2D, Supplementary Table 1). Ligand-induced thermal stability was also investigated using DSC with the catalytically inactive mutant E153A, which displayed unfolding transitions centered at 45.3 and 48.1 °C. The melting temperature (*T*_m_) values of E153A and *Sp*ChiD were nearly identical, indicating no great difference even after the point mutation. We therefore studied the effect of ligand binding on the thermal stability of E153A by pre-incubating the protein with CHOS of DP2–6 (Fig. 2E) and the thermodynamic parameters are presented in (Table 1). There was a considerable increment in the *T*_m_ of both transitions shown by E153A in presence of ligands DP2–6. However, the difference between *T*_m_ values of ligand bound to E153A and the protein alone (*i.e.* [*T*_m_ of E153A + (DP)_n_] − *T*_m_ of E153A alone), revealed that the second transition was more stable than the first transition with ligands DP2–4. Despite the high degree of stabilization when binding to DP5 (*T*_m1_ + 10.7 °C and *T*_m2_ + 11.2 °C) or DP6 (*T*_m1_ + 12.8 °C and *T*_m2_ + 12.4 °C), there was no substantial difference in the increment of *T*_m_ between transitions, after subtracting the corresponding *T*_m_ values of E153A alone. There were major differences in the *T*_m_ values upon ligand binding (Fig. 2E), which were accompanied by large increases in the microcalorimetric enthalpy *i.e.* Δ*H*_C._ This usually corresponds to the total amount of heat absorbed during unfolding and also the enthalpy driven interactions like ligand induced stabilization of the protein. Overall, the DSC data showed that the stability of *Sp*ChiD increased in the presence of ligands, an observation further supported by activity studies at different temperatures (Supplementary Fig. 1).

**Figure 3:**
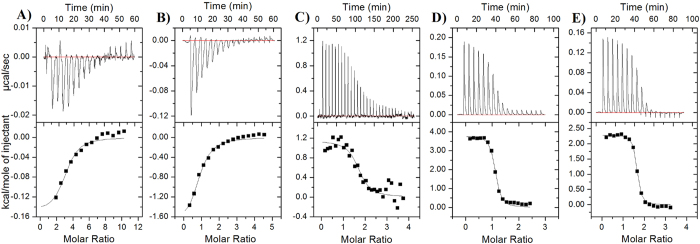
Binding properties of SpChiD-E153A assessed by ITC profiling. (**A**–**E**) ITC thermograms (upper panels) and binding isotherms with theoretical fits (lower panels) obtained for the binding of mutant E153A to CHOS with DP2–6, which represent the ligands (GlcNAc)_2-6_ unless otherwise specified.

Quenching studies with acrylamide, in the absence of ligand, suggested that mutation E153A did not cause major perturbations in the *Sp*ChiD tertiary structure. The percentage quenching values and Stern-Volmer quenching constants (*K*_SV_) for E153A, with and without CHOS (Fig. 2F, Supplementary Fig. 2, Supplementary Table 2), strongly suggested that the increase in ligand chain length reduced the accessibility of Trp to the quencher. ITC measurements showed that DP2, DP4 and DP6 bound to E153A with *n* values of 3.04, 1.69 and 1.58, respectively. In contrast, the *n* values for DP3 and DP5 were 0.84 and 1.09, respectively, indicating 1:1 stoichiometry. Analysis of the ITC data yields the equilibrium binding constant (*K*_d_) for ligands DP2 and DP3 to E153A was 0.2 μM, whereas DP4 displayed a *K*_d_ value of 0.093 μM. But the binding of ligands DP5 and DP6 resulted in very low *K*_d_ values of 0.013 and 0.005 μM, respectively, hitherto not reported for any family GH18 chitinase (NOTE: The binding constant (*K*_b_) values determined for *Sp*ChiD-E153A were converted to *K*_d_ in μM for a convenient comparison with the other GH18 or GH19 chitinases). ITC results indicate that the binding of CHOS (DP2−6) is driven predominantly by entropic forces. Although a minor enthalpic contribution was seen in the binding of shorter-chain CHOS (DP2 or DP3) as reflected in the exothermic heat changes in the ITC profiles (see Fig. 3A,B), the major contributor towards the binding process was entropic in nature (Table 2). On the other hand, binding of longer-chain CHOS (DP4, DP5 or DP6) was accompanied by strong endothermic heat changes, reflecting the negative contribution of binding enthalpy towards the association process (Fig. 3C–E).

### ‘Trp’ at the *Sp*ChiD substrate-binding cleft

**Figure 4:**
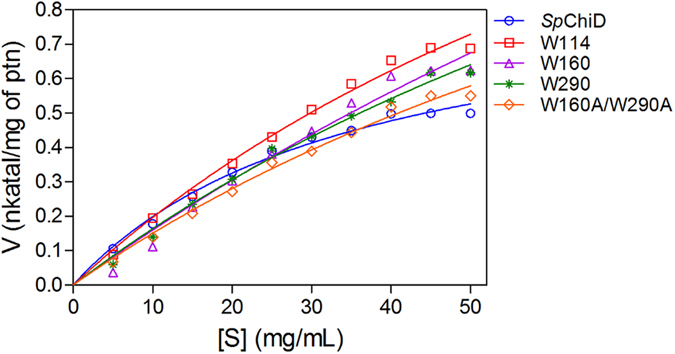
Kinetic analysis of the *Sp*ChiD Trp mutants. Different concentrations of colloidal chitin (0–50 mg/mL) were incubated with *Sp*ChiD and its Trp variants in 50 mM sodium phosphate buffer (pH 8.0) at 40 °C for 1 h at 200 rpm. Each reaction was carried out in triplicate along with appropriate controls. The specific activity (nkat/mg protein) was calculated and plotted against substrate concentration. The data were fitted to the Michaelis–Menten equation by nonlinear regression using GraphPad Prism v5.0, to obtain the kinetic graphs and parameters.

The fluorescence quenching and ligand binding data prompted us to evaluate the potential role of Trp residues at the substrate-binding cleft in the multiple functions of *Sp*ChiD. The high Michaelis−Menten constant (*K*_m_) values for the mutants W114A (105.1 mg/mL), W160A (208.9 mg/mL), W290A (133.6 mg/mL) and W160A/W290A (123.8 mg/mL) relative to *Sp*ChiD (35.1 mg/mL) indicated a reduction in affinity towards colloidal chitin (Fig. 4). The mutation of Trp residues also reduced the overall catalytic efficiency compared to *Sp*ChiD (Supplementary Table 3). These data provided strong evidence about the involvement of ‘Trp’ in binding to the polymeric substrate colloidal chitin.

**Figure 5:**
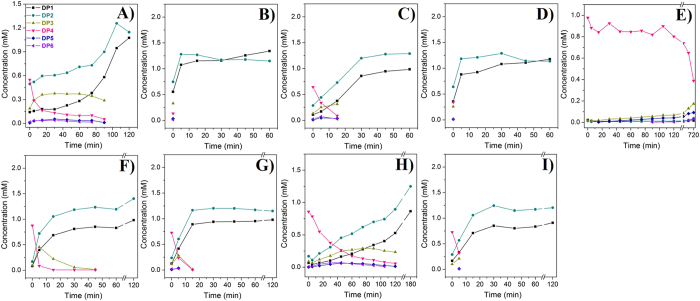
Product profiles of the *Sp*ChiD Trp mutants using DP4 substrate. HPLC profiles of (**A**) *Sp*ChiD and the Trp mutants at the substrate-binding cleft *i.e.* (**B**) W160A (**C**) W290A, (**D**) W160A/W290A and (**E**) W395A obtained by a linear correlation between peak area and concentration of oligosaccharide standards. HPLC analysis of the mutants (**F**) W114A (**G**) W160A (**H**) W290A and (**I**) W160A/W290A at low enzyme concentration confirmed the mutational effects. Individual quantification graphs represent all the hydrolytic (DP1–3) and TG (DP5, DP6) products that accumulated during the reaction with 1 mM DP4 substrate.

Mutant W160A showed an almost complete loss of TG activity but increased hydrolysis. TG activity was detected solely at 0 min and was minimal compared to *Sp*ChiD. After 5 min only DP1 and DP2 were generated, with proportions of 45.6 and 54.3%, respectively (Fig. 5A,B). Mutant W290A retained TG activity up to 15 min (Fig. 5C) and only DP1 and DP2 were present after 30 min, with proportions 43.3 and 56.6%, respectively. The double mutant, W160A/W290A also displayed very low TG activity only at 0 min and was minimal compared to *Sp*ChiD (Fig. 5D). Mutation W395A had a substantial negative impact on the rate of catalysis, which was evident from the slow decline in the initial concentration of DP4 substrate (Fig. 5E). W395A displayed TG activity, and was able to generate DP5 and DP6 after 15 and 90 min, respectively. This mutant produced more DP5 (12.4%) than DP6 (4.3%) at 720 min, and the total amount of quantifiable TG products (16.7%) exceeded the amount of DP1 + DP2 (7.2%). Much of the DP4 substrate (52.5%) was unchanged by mutant W395A even after 720 min. There was no loss of chitobiase activity in any of the Trp mutants.

To confirm the impact of the Trp mutations, the reactions were repeated using a five-fold lower concentration of the mutants W114A, W160A, W290A and W160A/W290A. Mutations W114A and W160A severely affected *Sp*ChiD TG activity but hydrolysis increased under these conditions (Fig. 5F,G). The mutant W290A produced more DP6 (6.3%) than DP5 (4.3%) at 30 min and displayed TG up to 120 min, but hydrolysis was predominant even with the low enzyme concentration (Fig. 5H). In the double mutant, the effect of W160A was dominant, with no TG activity detected even at the low enzyme concentration (Fig. 5I).

### Analysis of the loop region (Asn30-Asp42)

**Figure 6:**
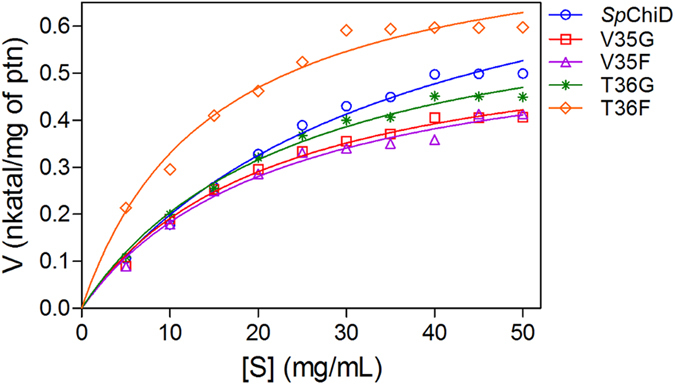
Kinetic analysis of the *Sp*ChiD loop variants. Different concentrations of colloidal chitin (0–50 mg/mL) were incubated with *Sp*ChiD and its loop variants in 50 mM sodium phosphate buffer (pH 8.0) at 40 °C for 1 h at 200 rpm. Each reaction was carried out in triplicate along with appropriate controls. The specific activity (nkat/mg protein) was calculated and plotted against substrate concentration. The data were fitted to the Michaelis–Menten equation by nonlinear regression using GraphPad Prism v5.0, to obtain the kinetic graphs and parameters.

**Figure 7:**
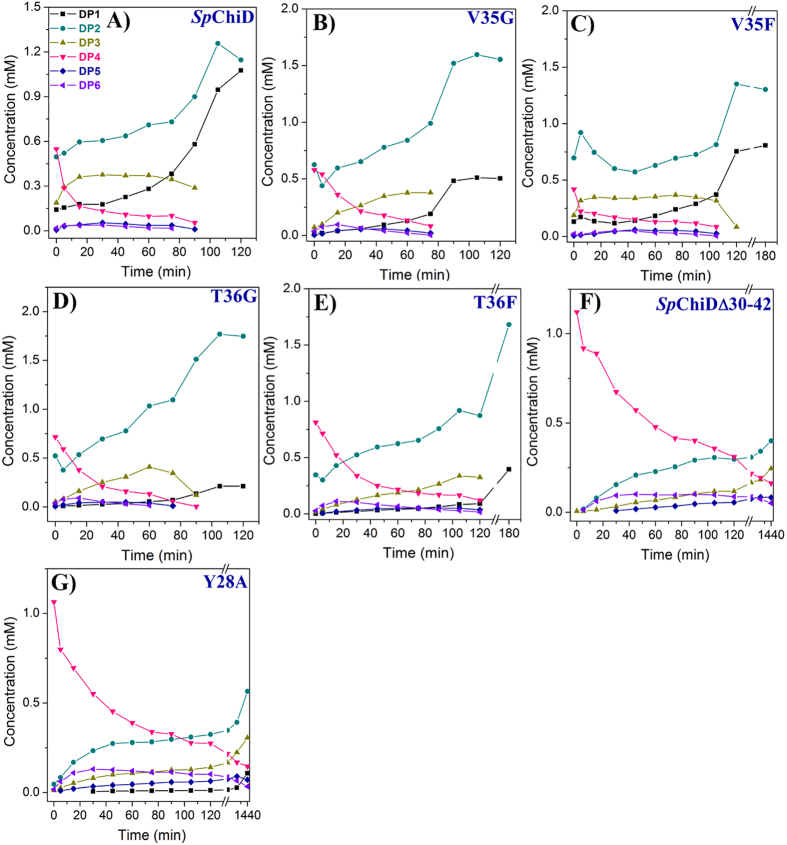
Product profiles for the *Sp*ChiD loop variants. HPLC profiles of *Sp*ChiD (**A**), Val variants (**B**,**C**), Thr variants (**D**,**E**), deletion mutant *Sp*ChiDΔ30–42 (**F**) and Y28A (**G**) obtained from the linear correlation between peak area and the concentration of oligosaccharide standards. Individual quantification graphs represent all the hydrolytic (DP1–3) and TG (DP5, DP6) products accumulated during the reaction with 1 mM DP4 substrate.

We analyzed enzyme kinetics for all the loop variants with colloidal chitin as a substrate (Fig. 6, Supplementary Table 4), and the changes in activity with DP4 substrate were monitored by high performance liquid chromatography (HPLC). Although the concentration of DP4 declined rapidly, the amount of DP1 and DP3 products produced by V35G at 30 min was low (4.6% and 20.2%, respectively) compared to *Sp*ChiD (12.7% and 27.2%, respectively) (Fig. 7A,B) and V35F (8.9% and 25.6%, respectively) (Fig. 7C). Almost equal quantities of DP2 were produced by both the Val variants. TG activity was retained up to 75 and 105 min by V35G and V35F, respectively, whereas, *Sp*ChiD displayed TG activity up to 90 min (Fig. 7A–C).

Of the two Thr variants, mutant T36F showed a low *K*_m_ of 14.7 mg/mL with a high overall catalytic efficiency of 63.5 s^−1^ mg^−1^ mL (Supplementary Table 4). But, DP2 was accumulated up to 89.2% after 120 min (T36G) and 81% after 180 min (T36F) (Fig. 7D,E). Both the Val and Thr variants produced low amounts of DP1 from DP4 substrate, suggesting there was a major impact on *Sp*ChiD chitobiase activity. In turn, deletion of the entire loop (Asn30–Asp42) reduced the rate of DP4 hydrolysis and abolished chitobiase activity (Fig. 7F). The reaction was monitored for 24 h, and the final ratio of products was 42.4% DP2, 26% DP3, 17.3% DP4, 8.8% DP5 and 5.4% DP6 (Fig. 7F). Furthermore, the mutant Y28A had displayed lower hydrolysis activity and a concomitant increase in the TG activity compared to *Sp*ChiD (Fig. 7G). The reaction was monitored for 24 h, and the final ratio of products was 8.7% DP1, 46% DP2, 24.8% DP3, 11.8% DP4, 5.8% DP5 and 2.8% DP6. Though DP1 was detected in the fractions, DP2 was the major end product even after 24 h. Thus, all the loop variants and the mutant Y28A produced no/low quantities of DP1 from the substrate DP4, suggesting a great impact on chitobiase activity of *Sp*ChiD.

**Figure 8:**
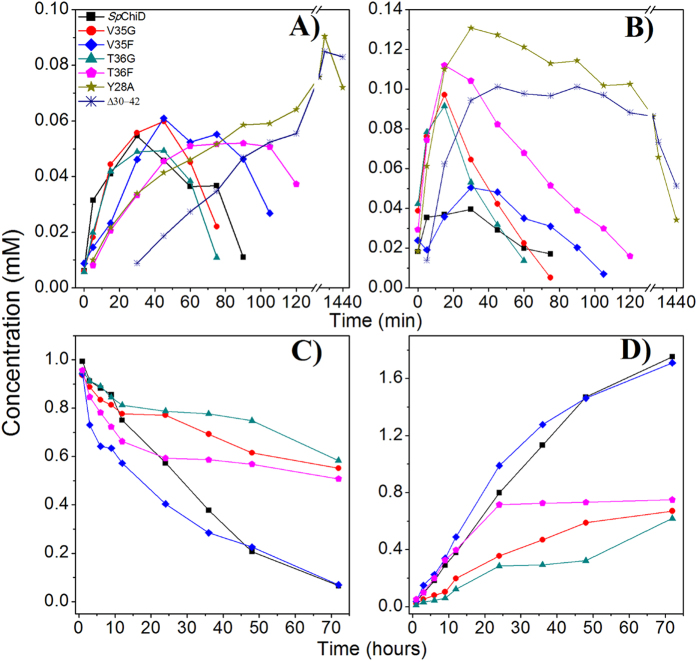
Comparison of quantifiable TG products and DP2 degradation abilities of *Sp*ChiD loop variants. The quantity of (**A**) DP5 and (**B**) DP6 TG products generated by *Sp*ChiD and its variants with 1 mM DP4 substrate were compared to the mutational effects on TG. The effect of mutations on chitobiase activity was monitored as a reduction in the amount of DP2 (**C**) and a concomitant increase in amount of DP1 (**D**). All reactions were carried out in 20 mM sodium acetate (pH 5.6) at 40 °C. The fractions collected at regular intervals were analyzed by measuring the absorption at 210 nm by isocratic HPLC on an Amino-P50 4E column. The product was quantified by linear correlation between the peak area and concentration of oligosaccharides in standard samples.

Due to a negative impact on chitobiase activity, both the Val variants generated more of DP6 than DP5 and the amount of DP6 produced by V35G (6.4%) at 5 min was six times greater than V35F (1.1%) (Fig. 8A,B). T36G produced more DP6 from 0 min, with a high proportion at 5 min (6.8%), followed by a gradual decline in the concentration of DP6. The formation of DP5 started slowly at 0 min, but reached 3.8% by 30 min. T36F produced DP5 after 5 min and reached a maximum of 4.3% by 60 min. DP6 accumulated to 9% by 30 min, which was three times more than *Sp*ChiD (2.8%), and twice the amount produced by T36G (4.2%). The TG activity was retained up to 75 and 120 min by T36G and T36F, respectively (Fig. 8A,B). In case of loop deletion mutant *Sp*ChiDΔ30−42, TG activity was increased to a greater extent with more DP6 accumulating than DP5 up to 180 min. After 30 min, the proportions of DP5 and DP6 products were 0.9% and 9.8%, respectively. A slow increase in the DP5 product started from 60 min (3%) and reached a maximum of 9.7% at 480 min (Fig. 8A). The proportions of DP6 produced by *Sp*ChiDΔ30−42, after 30, 60, 120 and 480 min were 9.8, 10.9, 10.1 and 8.4%, respectively (Fig. 8B). The concentration of DP6 (12.6%) generated by the mutant Y28A was four times higher than DP5 (3.2%) after 30 min. DP5 initially accumulated slowly but reached a proportion of 9.3% after 480 min, whereas the proportion of DP6 at 60, 120 and 480 min was 12.7%, 11.2% and 6.8%, respectively (Fig. 8A,B).

We investigated DP2 degradation to characterize all the loop mutants in more detail. The mutants *Sp*ChiDΔ30–42 and Y28A had completely lost the chitobiase activity. Among the Val and Thr variants, the chitobiase activity of V35F was unaffected, but there was a strong impact on DP2 degradation in mutant V35G and both Thr variants (Fig. 8C,D). The proportion of DP1 produced by *Sp*ChiD after 72 h was 96.4%, whereas the mutants V35G, V35F, T36G and T36F produced 54.8%, 96%, 51.4% and 59.5%, respectively. These data suggested the importance of residues Tyr28, Val35, Thr36 and the entire loop in chitobiase activity of *Sp*ChiD.

## Discussion

*Sp*ChiD displayed an exceptionally high TG activity, in addition to its other inherent functions, and this can be exploited to increase the yield of long-chain CHOS for biological applications. We therefore investigated the interplay between the multiple functions of *Sp*ChiD, controlled by the well-ordered substrate binding cleft. Previous DSC-based investigations of the changing heat capacity associated with the unfolding of multi-domain psychrophilic and mesophilic chitinases revealed the presence of a single unfolding transition^21^,^22^, whereas unfolding thermogram of the single-domain *Sp*ChiD protein is composed of two transitions. A similar double transition was earlier reported for a multi-modular family GH20 chitobiase, from *Arthrobacter* sp. strain TAD20 (*Ar*Chb), but, transition-1 was assigned to catalytic domain and transition-2 to the galactose-binding domain^23^, whereas, *Sp*ChiD was unique with its dual transitions confined to the single catalytic domain. Thus, it appears that the transition-1 corresponds to the partially unfolded intermediate state and transition-2 to the fully unfolded state of *Sp*ChiD. Inspite of its dual transitions, the apparent *T*_m_ of mesophilic *Sp*ChiD (*T*_m1_ = 50.1 °C and *T*_m2_ = 52.7 °C) was closer to the *T*_m_ of psychrophilic chitinases such as *Ar*ChiA (54.3 °C) and *Ar*ChiB (54 °C) from *Arthrobacter* sp. strain TAD20, and Chi60 (56.4 °C) from *Moritella marina* (*Mm*Chi60), and was significantly lower than the *T*_m_ of mesophilic *Sm*ChiA (64.2 °C).

The catalytic efficiency of enzymes acting at low temperatures has been attributed to their increased flexibility, reflecting the content of Arg, Pro and Gly residues^24^,^25^,^26^. The lower Arg content (2.5%) seems to contribute to the flexibility of the *Mm*Chi60 overall protein, in line with this observation, *Sp*ChiD also displayed a lower Arg content (3.4%), but similar amounts of Pro (4.9%) and Gly (8.8%)^22^. Since the substitution of Ala82 with Pro, in a lysozyme, increased its thermal stability^25^, a higher Ala content is potentially related to thermal lability. Thus, the high Gly and Ala but low Arg content of *Sp*ChiD may contribute to its flexibility and thermal lability, allowing the conformational changes necessary for hyper-TG activity.

So far three chitinases, two belonging to family GH18 (ChiB from *S. marcescens*−*Sm*ChiB and a chitinase from the insect *Ostrinia furnacalis*−*Of*ChtI) and one belonging to family GH19 (ChiA from moss species, *Bryum coronatum*−*Bc*ChiA) were studied in great detail for their binding properties using ITC^27^,^28^,^29^. Among these three chitinases, *Sm*ChiB displayed higher binding affinities towards DP4−6 CHOS (Table 3). We report for the first time the binding of DP2 to a family GH18 TG chitinase like *Sp*ChiD with a *K*_d_ value of 0.24 μM. The binding affinity for DP2 by *Sp*ChiD was nearly 3 times higher than DP5 binding to *Sm*ChiB (*K*_d_ = 0.67 μM), indicating a very tight binding of the second major end product of *Sp*ChiD mediated chitin hydrolysis. However, the binding affinity of E153A for DP3 was 1,515 fold higher when compared to *Sm*ChiB. The binding affinities of DP5 and DP6 to the mutant E153A were 52 and 26 times higher, respectively, than observed for *Sm*ChiB. The high binding affinities of the mutant E153A even for the lower chain CHOS like DP2 and DP3, indicates the probability of these CHOS getting transglycosylated at the substrate-binding cleft. Increased binding affinity with an increase in the chain length of CHOS was observed for the mutant E153A. A similar trend was observed for the *T*_m_ values obtained upon DP2−6 binding to E153A using DSC. Therefore, the exceptionally high binding affinities of the mutant E153A for CHOS with DP2−6 compared to any other GH18 or GH19 chitinases^27^,^28^,^29^ (Table 3) might be crucial to facilitate the unprecedented hyper-TG activity by *Sp*ChiD.

Electrostatic interactions are exothermic in nature and are stabilized at low temperatures, in contrast, hydrophobic interactions are endothermic and are weakened at lower temperatures^23^. Accordingly, ITC titrations with DP2 or DP3 were successful only at the lower temperature (13 °C) and were driven by exothermic heat change. In turn, titrations with DP5 or DP6 were successful at 25 °C, with a concomitant endothermic heat change. All the reactions were entropically driven with small enthalpic penalties, the important factor for this could be the topology and the architecture of binding site in *Sp*ChiD, designed for modulating both hydrolytic and TG activities. The binding of DP2/DP3 oligomers may depend more on electrostatic interactions than DP5/DP6 oligomers, which are more flexible and may therefore be involved in hydrophobic stacking interactions with the aromatic residues at the substrate-binding cleft. Accordingly, our fluorescence quenching data revealed that the accessibility of Trp residues at the binding cleft decreased to a greater extent in the presence of longer-chain CHOS such as DP5 and DP6. Therefore, Trp residues at the substrate-binding cleft appear to increase the affinity of substrate binding, thus influencing the TG activity of *Sp*ChiD. Mutational analysis also confirmed that Trp114, Trp160 and Trp290 are necessary for TG, and that Trp114 and Trp160 are the most important. The feeble TG activity displayed by the mutant W290A was lost when it was combined with the mutant W160A. The lower hydrophobicity of the binding cleft in these Trp mutants may facilitate the increased trafficking of water molecules along with CHOS and thus favor hydrolysis over TG activity. The high *K*_m_ values on colloidal chitin for all the Trp mutants also suggests the role of these residues in substrate binding. In mutant W395A, the loss of activity against colloidal chitin and the lower catalytic efficiency against DP4 substrate strongly support the role of Trp395 in *Sp*ChiD-mediated catalysis.

Many chitinases with feeble or no TG activity were previously shown to bind CHOS with a DP > 2 and therefore no chitobiase activity was observed^27^,^28^,^29^. The *Aspergillus niger* GH18 chitinase, CfcI can release monomers from CHOS with DP3–6, but cannot hydrolyze DP2 substrate^30^. In contrast, *Sp*ChiD demonstrated chitobiase activity in addition to its hydrolysis and TG activities^14^, a characteristic of GH20 enzymes. The ability of *Sp*ChiD to bind DP2 may favor chitobiase activity over TG activity, whereas perturbations in the positioning or binding of DP2 for chitobiase activity may increase the number of available DP2 molecules at the *Sp*ChiD substrate-binding cleft and thus increase TG activity. Residues Val35 and Thr36 appear to be necessary for chitobiase activity based on their ability to process DP4 and DP2 substrates. More importantly, the polar nature of Thr36 may promote interactions with DP2 and thus favor chitobiase activity, rather than the aromaticity conferred by the mutant Phe36.

*Sp*ChiD mutant Y28A displayed a decreased rate of hydrolysis with DP4 substrate, similar to the Y10F mutant of *Sm*ChiB^9^,^11^, but this was also accompanied by enhanced TG activity. The Y28A mutation also resulted in a complete loss of chitobiase activity. Perturbations in the charge stabilizing role played by the residue Tyr28^9^ may prolong the retention of the substrate or the intermediate oxazoline at the substrate-binding cleft and thus promote TG activity. *Sp*ChiD is active against polymeric substrates^14^, which may depend on the flexible nature of the Asn30-Asp42 loop at the binding cleft. Deletion of the entire loop or the mutation Y28A not only affected *Sp*ChiD chitobiase activity, but also the hydrolytic activity against chitin polymers. However, these mutants produced greater quantities of even-chain TG products, especially the quantifiable TG product DP6, indicating an inverse relationship between the chitobiase and TG activities of *Sp*ChiD.

**Figure 9:**
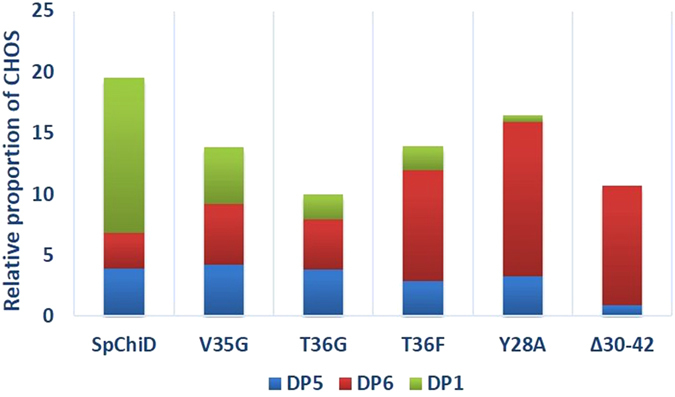
Overview of the products generated by *Sp*ChiD and selected mutants. The quantity of *N*-acetyl glucosamine and TG products (DP5 and DP6) generated by *Sp*ChiD and its variants with 1 mM DP4 substrate. The effect of mutations on chitobiase activity and simultaneous increase in TG was monitored as a reduction in the amount of *N*-acetyl glucosamine and a concomitant increase in amount of DP5/DP6. The products were quantified by linear correlation between the peak area and concentration of respective CHOS in standard samples.

We summarize in Fig. 9 that *N*-acetyl glucosamine was the major end product indicative of the chitobiase activity of *Sp*ChiD. But, in the Val or Thr or Tyr mutants and/or when the loop was deleted, there was a significant or total loss of chitobiase activity so that the proportion of *N*-acetyl glucosamine was low or nil. In these mutants, we also observed a significant increase in the TG products, particularly chitohexaose (even chain CHOS *i.e.* DP6), suggesting that there was an inverse relation between the chitobiase and TG activities of *Sp*ChiD. Further, we conclude that the substrate-binding cleft of a chitinase like *Sp*ChiD regulates multiple activities, and could therefore play a similar vital role in the 17 other chitinases present in the same phylogenetic cluster. The present study provides a better understanding of the diverged substrate-binding cleft of *Sp*ChiD. In addition, the hyper TG mutant Y28A, reported here, can be employed in enzyme-based bio-processes for the production of longer chain CHOS.

### Materials and Methods

#### Generation of *Sp*ChiD mutants and heterologous expression

*Sp*ChiD mutants were generated using pET 22b*-Sp*ChiD as template, essentially as previously described^18^. Mutagenic primers used in the present study are listed in Supplementary Table 5. *Escherichia coli* Rosetta-gami II (DE3) cells containing the appropriate *Sp*ChiD mutants were used for protein overexpression as previously described^14^.

### Protein isolation and Ni-NTA purification

Periplasmic fractions were prepared using the two-step osmotic shock procedure described in the pET expression system instructions (Novagen) with minor modifications. The supernatant was passed through a 0.2-μm sterile filter before purification. The periplasmic fraction was buffer exchanged against lysis buffer (50 mM NaH_2_PO_4_, 300 mM NaCl, 10 mM imidazole, pH 8.0), which was also used as the equilibration buffer in further affinity purification steps^14^. The fractions were separated by 12% SDS-PAGE to check the purity of the protein.

### Zymogram and kinetic analysis

The activity of purified *Sp*ChiD mutants was determined by dot blot zymography. A composite gel supplemented with 0.1% glycol chitin was prepared, and 5 μg each of purified chitinase was spotted on to the gel and placed in humid chamber at 37 °C overnight. The gel was stained with 0.01% calcofluor white M2R in 0.5 M Tris-HCl (pH 8.8) for 10 min at 4 °C. Finally, the brightener solution was removed, and the gel was washed with double-distilled water for 30 min at 4 °C. Lytic zones were visualized as dark blue spots when the gels were observed on a UV transilluminator. Kinetic parameters for all *Sp*ChiD mutants were determined by incubating 38 μg of each enzyme with different concentrations of colloidal chitin (0–50 mg/mL) in 50 mM sodium phosphate, pH 8.0. Proper controls for buffer, enzyme and substrate alone were maintained. All the reactions were carried out at 40 °C for 1 h, with constant shaking at 200 rpm. Chitinase activity was measured as described previously^31^ and was defined as the release of one micromole of GlcNAc per second under standard experimental conditions. Specific activity was calculated in nkat/mg of protein. Kinetic values were obtained by fitting the average of three independent sets of data to the Michaelis−Menten equation by nonlinear regression in GraphPad Prism v5.0 (GraphPad Software Inc., San Diego, CA).

### High performance liquid chromatography (HPLC)

We incubated 1 mM substrate (DP4 or DP2) with 250 nM purified wild-type or mutant *Sp*ChiD at 40 °C in 20 mM sodium acetate (pH 5.6). Fractions were collected at regular intervals and the reaction was stopped using an equal volume of 70% acetonitrile. The 20-μL reaction mixture from each fraction was resolved on a SHODEX Amino-P50 4E column (4.6 ID × 250 mm, Showa Denko K.K, USA) by isocratic elution with 70% acetonitrile at a flow rate of 0.7 mL/min, and the absorbance was monitored at 210 nm. CHOS (DP1–6) were quantified as previously described^18^.

### Circular dichroism (CD) spectroscopy

CD spectra were obtained on Jasco J-810 spectropolarimeter (Jasco International Co. Ltd, Tokyo, Japan) fitted with rectangular 0.7-mL cuvettes (path length 0.2 cm) using 0.06 and 0.6 mg/mL samples of *Sp*ChiD for the far and near UV regions, respectively. Measurements were taken within the temperature range 30–50 °C (Peltier heating system) at a scan rate of 20 nm/min, with a response time of 4 s and a slit width of 2 nm. A good signal-to-noise ratio was obtained by averaging five consecutive scans collected from the 190–260 nm far UV and 260–350 nm near UV ranges at 1-nm intervals. Buffer scans recorded under identical conditions were used as blanks.

### Differential scanning calorimetry (DSC)

DSC measurements were carried out on a MicroCal VP-DSC (MicroCal LLC, Northampton, MA, USA). *Sp*ChiD and mutant E153A were dialyzed against 20 mM potassium phosphate buffer, pH 8.0. The dialysate was used as the reference. Protein samples (0.5 mg/mL) were heated from 10–70 °C at a scan rate of 30°/h to assess the effect of ligand binding on thermal unfolding. Mutant E153A was pre-incubated for 1 h with 1 mM of each ligand (DP2–6) before scanning. Sample and reference solutions were degassed prior to scanning to eliminate bubbling effects. Baseline reproducibility was verified by multiple scans, and the reversibility of protein unfolding was monitored by scanning the sample twice. Buffer scans were subtracted from thermograms obtained in the presence or absence of CHOS before further analysis^32^. Absolute and excess heat capacities were analyzed using a non-two-state fit of the thermogram in Origin v7.0.

### Fluorescence spectroscopy

The accessibility of Trp residues in the presence and absence of CHOS was studied by acrylamide quenching. Mutant E153A (0.06 mg/mL) was pre-incubated with 1 mM of each ligand (DP2–6) and titrated with 5 M acrylamide in 20 mM potassium phosphate buffer, pH 8.0. A titration with 1 mM ligand in the same buffer was used to detect non-specific changes in relative fluorescence intensity due to ionic-strength variation. Fluorescence spectra were corrected for buffer subtraction and volume changes before further analysis^32^. Quenching data were analyzed according to the Stern-Volmer equation (F_O_/F = 1 + *K*_SV_ [Q]) where F_O_ and F are the fluorescent intensities in the absence and presence of the quencher, respectively, *K*_SV_ is the Stern-Volmer quenching constant and [Q] is the molar quencher concentration^33^.

### Isothermal titration calorimetry (ITC)

Calorimetric titrations were carried out at 13 °C with DP2–4 CHOS, and at 25 °C with DP5 and DP6 CHOS, using a MicroCal ITC_200_ System (Microcal, Northampton, MA, USA). Mutant E153A was extensively dialyzed against 20 mM potassium phosphate (pH 8.0) and the dialysate was used to make CHOS solutions to minimize the heat of dilution during titration. Protein solutions were passed through 0.2-μm filters and thoroughly degassed prior to use. Mutant E153A was titrated by injecting 2-μL aliquots of each CHOS ligand, while maintaining the enzyme concentrations at 113 μM (the ligand concentration was 2–6 mM). Successive injections at 225-s intervals ensured that equilibrium was achieved before adding the next aliquot. The sample in the calorimeter cell was stirred at 260 rpm during titration. Blank titrations were also performed by injecting ligand alone into the buffer solution, which yielded insignificant heat of dilution values. The binding isotherms were analyzed using MicroCal Origin v7.0. Thermodynamic parameters such as binding stoichiometry (n), change in enthalpy (Δ*H*) and association constant (*K*a) were obtained by nonlinear least-squares fitting of experimental data using the one-set-of-sites binding model^34^. Free energy of binding (Δ*G*) and change in entropy (Δ*S*) were deduced from the *K*a and Δ*H* values using the standard thermodynamic equation


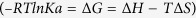


### Multiple sequence alignment (MSA)

The *Sp*ChiD sequence comprising the catalytic TIM barrel domain (PDB: 4LGX) was aligned with the catalytic domains of chitinases *Kp*ChiII (PDB: 3QOK), *Sm*ChiA (PDB: 1CTN) and *Sm*ChiB (PDB: 1E15) using ClustalW2 (www.ebi.ac.uk/Tools/msa/clustalw2/). *Sp*ChiD was also aligned with all the single-domain GH18 chitinases from the Enterobacteriaceae and some of the well-characterized chitinases from *Bacillus thuringiensis, B. licheniformis, B. circulans, S. marcescens, Stenotrophomonas maltophilia* and *Flavobacterium johnsoniae*.

### Phylogenetic analysis

The phylogenetic relationship among the sequences was inferred using the maximum likelihood method based on the Jones-Taylor-Thornton (JTT) matrix-based model^35^. The bootstrap consensus tree inferred from 500 replicates was taken to represent the evolutionary history of the taxa^36^. Branches corresponding to partitions reproduced in less than 50% bootstrap replicates were collapsed. Initial tree(s) for the heuristic search were obtained by applying the neighbor-joining method to a matrix of pairwise distances estimated using a JTT model. The analysis involved 131 amino acid sequences representing bacterial family GH18 chitinases (abbreviations and enzyme sources are listed in Supplementary Table 6). The positions containing gaps and missing data were eliminated leaving 70 positions in the final dataset. The phylogenetic analysis was carried out using MEGA v6^37^.

## Additional Information

**How to cite this article**: Madhuprakash, J. *et al.* Inverse relationship between chitobiase and transglycosylation activities of chitinase-D from *Serratia proteamaculans* revealed by mutational and biophysical analyses. *Sci. Rep.* **5**, 15657; doi: 10.1038/srep15657 (2015).

## Supplementary Material

Supplementary Information

## Figures and Tables

**Figure 1:**
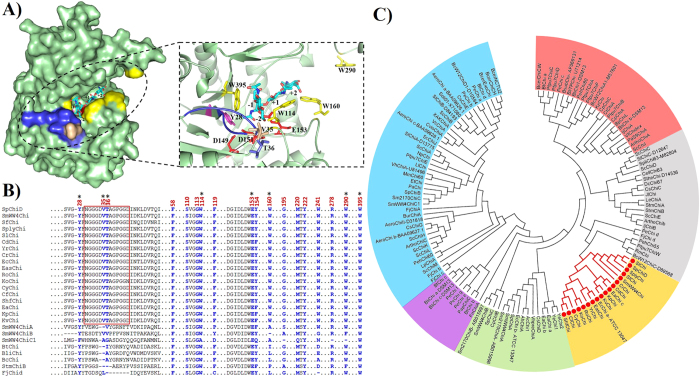
The substrate-binding cleft of SpChiD, phylogenetic analysis and its evolutionary divergence. (**A**) Surface representation of *Sp*ChiD (PDB: 4LGX) showing the substrate-binding cleft (dashed circle) and the ligand DP4 at the active site (stick with carbon atoms in cyan). Magnified image in the dashed box represents the stereoview of the substrate-binding cleft showing catalytic residues (red), Trp (yellow), Tyr (pink), Val (gray), Thr (pale blue) and the entire loop region Asn30–Asp42 (blue). The numbers indicate the probable subsites to which the ligand can bind. (**B**) Multiple sequence alignment showing the novel loop (Asn30–Asp42) identified in *Sp*ChiD (rectangular box), is highly conserved among 18 different single-domain chitinases from Enterobacteriaceae, showing their divergence from other groups. The residues so far analyzed through mutagenesis in *Sp*ChiD^18^,^19^ (highlighted in blue) were conserved in all these single-domain GH18 chitinases with a loop. The residues analyzed in the present study are indicated with an asterisk. (**C**) Phylogenetic relationship between *Sp*ChiD and 131 bacterial family-GH18 sequences. The unrooted phylogenetic tree revealed that with the exception of three chitinases (*Ye*Chi1, *Ye*Chi2 and *Ef*Chi), all the single-domain GH18 sequences from the family Enterobacteriaceae related to *Sp*ChiD clustered into a separate group (highlighted in yellow). The abbreviations and enzyme sources with accession numbers for this phylogenetic tree are listed in Supplementary Table 6.

**Table 1:** DSC-determined transition temperatures (*T*_m_) of thermal unfolding and the thermodynamic parameters of CHOS binding to E153A.

	***T***_**m**_ **(°C)**		**Δ*****H***_**C**_**(kcal.mol**^**−1**^)		**Δ*****H***_**v**_**(kcal.mol**^**−1**^)		**Δ*****H***_**C**_**/Δ*****H***_**v**_	
	**Transition-I**	**Transition-II**	**Transition-I**	**Transition-II**	**Transition-I**	**Transition-II**	**Transition-I**	**Transition-II**
E153A	45.34 ± 0.18	48.12 ± 0.028	136.1 ± 9.87	121 ± 9.29	132.4 ± 3.44	250.1 ± 9.16	1.02	0.48
DP2	48.78 ± 0.03	53.16 ± 0.096	798.5 ± 7.14	22.03 ± 4.1	106.5 ± 1.07	499.5 ± 92.1	7.49	0.04
DP3	50.20 ± 0.47	54.30 ± 0.043	395.1 ± 49.3	556.6 ± 46.4	87.17 ± 3.71	165.3 ± 6.23	4.53	3.36
DP4	48.88 ± 0.52	54.10 ± 0.032	361.6 ± 38.7	643.0 ± 35.0	70.95 ± 3.63	153.2 ± 4.33	5.09	4.19
DP5	56.09 ± 0.30	59.30 ± 0.063	537.9 ± 61.8	500.5 ± 59.8	123.8 ± 3.94	203.4 ± 10.2	4.34	2.46
DP6	58.15 ± 0.19	60.49 ± 0.055	379.6 ± 36.7	234.5 ± 35.2	176.8 ± 4.67	306.9 ± 22.1	2.14	0.76

**Table 2:** ITC-based thermodynamic parameters of CHOS binding to the *Sp*ChiD mutant E153A. DP2–6 represent the ligands (GlcNAc)_2–6_ unless otherwise specified (*K*_b_ − binding constant).

**Sugar**	**Temp**	***n***	***K***_**b**_ ** × 10**^**5**^ **(M**^**−1**^)	**−Δ*****G*****° (kcal.mol**^**−1**^)	**Δ*****H*****° (kcal.mol**^**−1**^)	**Δ*****S*****° (cal.mol**^**−1**^**.deg**^**−1**^)	**−*****T*****Δ*****S*** **kcal.mol**^**−1**^
DP2	13 °C	3.04 ± 0.307	0.422 ± 0.207	6.04	−0.1506 ± 0.0261	20.6	5.89
DP3	13 °C	0.839 ± 0.0641	0.461 ± 0.117	6.1	−1.837 ± 0.186	14.9	4.26
DP4	13 °C	1.69 ± 0.071	1.07 ± 0.53	6.6	1.148 ± 0.0675	27	7.72
DP5	25 °C	1.09 ± 0.00966	7.48 ± 1.18	8	3.530 ± 0.0468	38.7	11.53
DP6	25 °C	1.58 ± 0.00962	17.9 ± 3.67	8.53	2.281 ± 0.0249	36.3	10.81

**Table 3:** Dissociation constants (*K*_d_ values in μM) of CHOS (DP2–6) to the catalytically-inactive mutants of two GH18 chitinases (*Sm*ChiB-E144Q and *Of*ChtI-E148Q)^27^,^28^ and one GH19 chitinase (*Bc*ChiA-E61A)^29^ were compared against *Sp*ChiD-E153A. Low *K*_d_ values for *Sp*ChiD-E153A binding to CHOS indicate exceptionally high binding affinities hitherto not reported for any chitinase.

**Ligand**	***Sp*****ChiD-E153A**	***Sm*****ChiB-E144Q**	***Of*****ChtI-E148Q**	***Bc*****ChiA-E61A**
DP2	0.237	NR	NR	NR
DP3	0.217	327 ± 22	*K*_d1_:76.9 ± 18.9 *K*_d2_:284.1 ± 97.4	230 ± 20
DP4	0.093	2.7 ± 0.19	11.3 ± 1.5	16 ± 1
DP5	0.013	0.67 ± 0.20	2.0 ± 0.2	1.8 ± 0.3
DP6	0.005	0.13 ± 0.09	0.48 ± 0.09	0.77 ± 0.06

NR: not reported.
