# Supplementary material for: Inverse relationship between chitobiase and transglycosylation activities of chitinase-D from Serratia proteamaculans revealed by mutational and biophysical analyses
Source: Sci Rep. 2015 Oct 23;5:15657. doi: 10.1038/srep15657 (PMC4616163; doi:10.1038/srep15657)
Supplement: Supplementary Information [file srep15657-s1.pdf]

Inverse relationship between chitobiase and transglycosylation activities of chitinase-D from *Serratia proteamaculans* revealed by mutational and biophysical analyses

Jogi Madhuprakash[1], Kishore Babu Bobbili[2], Bruno M Moerschbacher[3], Tej Pal Singh[4], Musti J. Swamy[2] and Appa Rao Podile*[1]

[1]Department of Plant Sciences, School of Life Sciences, University of Hyderabad, Gachibowli, Hyderabad, India,
[2]School of Chemistry, University of Hyderabad, Gachibowli, Hyderabad, India,
[3]Institute for Biology and Biotechnology of Plants, WWU, Münster University, Münster, Germany,
[4]Department of Biophysics, All India Institute of Medical Sciences, New Delhi, India

***Author for correspondence:**
Prof. Appa Rao Podile
Department of Plant Sciences
School of Life Sciences
University of Hyderabad
Gachibowli,
Hyderabad-500046, India
Tel: +91-40-23134503
Fax: +91-40-23010120
E-mail: arpsl@uohyd.ernet.in

pH	T_{m1} (°C)	ΔHc_1 (kcal mol^{-1})	ΔHv_1 (kcal mol^{-1})	$\Delta Hc_1/\Delta Hv_1$	T_{m2} (°C)	ΔHc_2 (kcal mol^{-1})	ΔHv_2 (kcal mol^{-1})	$\Delta Hc_2/\Delta Hv_2$
2	42.55	81.14	126.9	0.639	45.18	64.83	204.1	0.317
4	47.86	134.0	143.0	0.937	50.22	118.0	238.8	0.494
6	50.14	101.5	132.4	0.766	52.76	65.80	234.4	0.280
8	44.87	157.1	138.2	1.13	47.29	100.5	227.3	0.442
10	41.51	108.9	138.6	0.785	44.23	76.14	207.0	0.367

Table S1: DSC-determined calorimetric enthalpy (ΔHc), Van't Hoff enthalpy (ΔHv) and their ratio (ΔHc/ΔHv) corresponding to the transitions shown by *Sp*ChiD at different pH values.

	% Quenching	*K*sv
*Sp*ChiD	82.4	9.17
E153A	83.9	10.09
DP2	66.5	3.95
DP3	65.8	3.73
DP4	61.1	3.33
DP5	60.2	2.91
DP6	54.7	2.41

Table S2: Acrylamide quenching of E153A in the presence or absence of ligands DP2–6.

Enzyme	K_m (mg/mL)	V_{max} (nkat/mg of protein)	K_{cat} (s^{-1})	K_{cat}/K_m (s^{-1} mg^{-1} mL)
*Sp*ChiD	35.12	0.896	10.3×10^2	29.33
W114A	**105.1**	2.262	25.99×10^2	24.73
W160A	**208.9**	3.494	40.14×10^2	19.22
W290A	**133.6**	2.352	27.02×10^2	20.23
W160A/W290A	**123.8**	2.014	23.14×10^2	18.69
W395A*	ND	ND	ND	ND

Table S3: Kinetic parameters of Trp variants at the substrate binding cleft. *Kinetic parameters could not be determined because there was no activity against colloidal chitin.

Enzyme	K_m (mg/mL)	V_{max} (nkat/mg of protein)	K_{cat} (s^{-1})	K_{cat}/K_m (s^{-1} mg^{-1} mL)
*Sp*ChiD	35.12	0.896	10.3×10^2	29.33
V35G	21.7	0.605	6.95×10^2	32.06
V35F	22.73	0.598	6.88×10^2	30.26
T36G	24.19	0.697	8.01×10^2	33.13
**T36F**	**14.75**	**0.815**	$\mathbf{9.36\times10^2}$	**63.47**
Y28A*	ND	ND	ND	ND
Δ30-42*	ND	ND	ND	ND

Table S4: Kinetic parameters of loop variants at the substrate binding cleft. *Kinetic parameters could not be determined because there was no activity against colloidal chitin.

Mutant	Primer sequence
Y28A	5'–CTT TCC GTC GGT *GCC* TTC AAC GGT GGC–3'
V35G	5'–GGT GGC GGT GAT *GGT* ACC GCC GGT CCT–3'
V35F	5'–GGT GGC GGT GAT *TTC* ACC GCC GGT CCT–3'
T36G	5'–GGC GGT GAT GTT *GGC* GCC GGT CCT GGT–3'
T36F	5'–GGC GGT GAT GTT *TTC* GCC GGT CCT GGT–3'
E153A	5'–GAT CTC GAC TGG *GCA* TAC CCG GTT AAC–3'
W160A	5'–GTT AAC GGT GCC *GCG* GGA CTG GTC GAA–3'
W290A	5'–CCG GGC ATC GAT *GCG* GAC AAG GCG GAT–3'
W395A	5'–GGT GCG ATG TTC *GCG* GAA TAT GGC GCA–3'
Δ30-42 RpO	5'–GTC GAG CTT GTT GAT GAA ATA ACC GAC GGA–3'
Δ30-42 IFp	5'–ATC AAC AAG CTC GAC GTC ACA CAA ATT ACC–3'

Table S5: Primers used for *Sp*ChiD mutagenesis. Underlined sequences encode the substituted amino acid. Mutants prefixed with Δ in the first column are deletion mutants.

Abbreviation	Enzyme and source	Accession No.
BcerChiCW	chitinase CW [Bacillus cereus]	AAM48520.2
BtChi a	Bacillus thuringiensis serovar kurstaki str. HD73	AGE76094.1
Pfpu7ChiC	Paenibacillus sp. FPU-7	BAM67139.1
Pfpu7ChiD	Paenibacillus sp. FPU-7	BAM67140.1
PeChi c	chitinase [Paenibacillus elgii]	WP_010499208.1
BsubChi-AF069131	chitinase [Bacillus subtilis]	AAC23715.1
BliTPChi- U71214	chitinase [Bacillus licheniformis]	AAB47847.1
BliChi1-DSM13	[Bacillus licheniformis DSM 13 = ATCC 14580]	AAU21943.2
PehChi80	chitinase Chi80 [Paenibacillus ehimensis]	BAC76694.1
Pfpu7ChiA	[Paenibacillus sp. FPU-7]	BAM67137.1
Pfpu7ChiF	[Paenibacillus sp. FPU-7]	BAM67142.1
BcW12ChiA1-M57601	chitinase A1 [Bacillus circulans]	AAA81528.1
KzChi	chitinase [Kurthia zopfii]	BAA09831.1
CpChiC	[Clostridium paraputrificum]	BAD12045.1
CtChiA	Chitinase [Clostridium thermocellum ATCC 27405]	CAA93150.1
Pfpu7ChiB	[Paenibacillus sp. FPU-7]	BAM67138.1
CpChiA	Chitinase A [Clostridium paraputrificum]	BAA34922.1
BpChiL	chitinase large [Bacillus cf. pumilus SG2]	ABF50676.1
BliChi b-DSM13	putative glycoside hydrolase [Bacillus licheniformis DSM 13 = ATCC 14580]	AAU39297.1
IjChiA	chitinase A [Isoptericola jiangsuensis]	ADD17350.1
VfChidex	chitodextrinase [Vibrio furnissii]	AAC44673.1
PaS9ChiA	chitinase A [Pseudoalteromonas sp. S9]	AAC79665.1
ArthoChiA	chitinase [Arthrobacter sp.]	CAB62382.1
ScChiI	putative chitinase precursor [Streptomyces coelicolor A3(2)]	CAB76866.1
ScChiC	chitinase C [Streptomyces coelicolor A3(2)]	CAB94547.1
SlChiC- D12647	chitinase C [Streptomyces lividans]	BAA02168.1
SpliChi63- M82804	chitinase 63 [Streptomyces plicatus]	AAA26720.1
ScChiD	Chitinase [Streptomyces coelicolor A3(2)]	CAB61662.1
Cellchi63	endo-63 precursor [Cellulomonas sp. GM13]	AAF00931.2
StheChi- D14536	Chitinase precursor [Streptomyces thermoviolaceus]	BAA03404.1
DcChi67	chitinase Chi67 [Doohwaniella chitinasigens]	AAF21468.1
CsChiC	[Chitiniphilus shinanonensis]	BAK53887.1
JlChi	[Janthinobacterium lividum]	AAA83223.1
LeChiA	[Lysobacter enzymogenes]	AAT77163.1
StmChiA	[Stenotrophomonas maltophilia]	CAQ44264.1
StmChiB	[Stenotrophomonas maltophilia]	CAQ46811.1
ScChiE	[Streptomyces coelicolor A3(2)]	CAA16211.1
ArthoChiB	[Arthrobacter sp.]	CAB62499.1
IjChiB	[Isoptericola jiangsuensis]	ADD17351.1
PeChi d	[Paenibacillus elgii]	WP_010502184.1
FjChi d	[Flavobacterium johnsoniae UW101]	ABQ07756.1
PehChi55	[Paenibacillus ehimensis]	BAC76692.1
Pfpu7ChiW	[Paenibacillus sp. FPU-7]	BAM67143.1
RmChi	[Rhodothermus marinus]	AAU11838.1
BcW12ChiC-D89568	[Bacillus circulans]	BAA13974.1

Table S6: List of abbreviations and enzyme sources with GenBank accession numbers.

Continued..

Abbreviation	Enzyme and source	Accession No.
SlChi	[Serratia liquefaciens ATCC 27592]	YP_008230673.1
SpChiD	[Serratia proteamaculans]	ABV41826.1
SplyChi	[Serratia plymuthica 4Rx13]	YP_008138745.1
SfChi	[Serratia fonticola]	WP_021181207.1
SmWW4Chi	[Serratia marcescens WW4]	AGE18533.1
CrChi	[Citrobacter rodentium ICC168]	YP_003364864.1
CdChi	[Cedecea davisae]	WP_016535963.1
EaChi c	[Enterobacter aerogenes KCTC 2190]	AEG99244.1
KpChi	[Klebsiella pneumoniae subsp. pneumoniae MGH 78578]	YP_001334864.1
KvChi	[Klebsiella variicola CAG:634]	WP_022065505.1
EcChi a- ATCC 13047	[Enterobacter cloacae subsp. cloacae ATCC 13047]	ADF62010.1
EasChi	[Enterobacter asburiae LF7a]	YP_004828221.1
YrChi	[Yokenella regensburgei]	WP_006819576.1
RoChi	[Raoultella ornithinolytica B6]	YP_007873975.1
KoChi	[Klebsiella oxytoca]	WP_004101274.1
CyChi	[Citrobacter youngae]	WP_006684883.1
CfChi	[Citrobacter freundii]	WP_003840873.1
ShfChi	[Shigella flexneri 1235-66]	EIQ80021.1
YeChi2	[Yersinia entomophaga]	ABG33867.1
YeChi1	[Yersinia entomophaga]	ABG33870.1
EaChi a	[Enterobacter aerogenes KCTC 2190]	AEG97068.1
EaChi b	[Enterobacter aerogenes KCTC 2190]	AEG97642.1
CsChiD	[Chitiniphilus shinanonensis]	BAK53888.1
VhpreChiA	[Vibrio harveyi]	AAK11576.1
AcChi1	[Aeromonas caviae]	AAA93130.1
AhChiA	[Aeromonas hydrophila]	AAF70180.1
EcChi b- ATCC 13047	[Enterobacter cloacae subsp. cloacae ATCC 13047]	ADF62328.1
SpChiA	Serratia proteamaculans	ABV39247.1
SmWW4ChiA	[Serratia marcescens WW4]	AGE15954.1
Sm2170ChiA-AB015996	[Serratia marcescens]	BAA31567.1
CpChiB	[Clostridium paraputrificum]	BAA23796.1
SpChiC	Serratia proteamaculans	ABV42574.1
SmWW4ChiB	[Serratia marcescens WW4]	AGE19379.1
Sm2170ChiB-AB015997	[Serratia marcescens]	BAA31568.1
FnChiA	[Francisella novicida]	ABB76136.1
BtChi b	[Bacillus thuringiensis serovar kurstaki str. HD73]	AGE79252.1
PapChiC	[Pseudoalteromonas piscicida]	BAA24795.1
PeChi e	[Paenibacillus elgii]	WP_010502184.1
PeChi g	[Paenibacillus elgii]	WP_010499339.1
PeChi f	[Paenibacillus elgii]	WP_010500806.1
BliChi c-DSM13	[Bacillus licheniformis DSM 13 = ATCC 14580]	AAU40846.1
BliChi a-DSM13	[Bacillus licheniformis DSM 13 = ATCC 14580]	AAU39010.1
BtChi c	[Bacillus thuringiensis serovar kurstaki str. HD73]	AGE79460.1

Table S6: List of abbreviations and enzyme sources with GenBank accession numbers.

Continued..

Abbreviation	Enzyme and source	Accession No.
FjChiC	[Flavobacterium johnsoniae UW101]	ABQ07559.1
FjChi b	[Flavobacterium johnsoniae UW101]	ABQ07183.1
ScChiM	[Streptomyces coelicolor A3(2)]	CAC16966.1
LpChiA	[Legionella pneumophila str. Lens]	CAH15360.1
PehChi60	[Paenibacillus ehimensis]	BAC76693.1
ScChiL	[Streptomyces coelicolor A3(2)]	CAC10108.1
ScChiK	[Streptomyces coelicolor A3(2)]	CAB44541.1
ArthoChiC	[Arthrobacter sp. TAD20]	CAD43215.1
ScChiH	[Streptomyces coelicolor A3(2)]	CAA15789.1
AeroChi b- BAA09627.1	[Aeromonas sp. 10S-24]	BAA09627.1
CsChiG	[Chitiniphilus shinanonensis]	BAK53891.1
AeroChiII-D31818	[Aeromonas sp. 10S-24]	BAA06605.1
BurChiA	[Burkholderia gladioli]	BAA92251.1
FjChiA	[Flavobacterium johnsoniae UW101]	ABQ07554.1
SmWW4ChiC1	[Serratia marcescens WW4]	AGE16995.1
Sm2170ChiC	[Serratia marcescens]	WP_021504616.1
SpChiB	[Serratia proteamaculans]	ABV40327.1
PaChi	[Pseudomonas aeruginosa PAO1]	AAG05688.1
EfChi	[Enterococcus faecalis V583]	AAO80224.1
MmChi60	[Moritella marina]	CAM88673.1
VhChiA- U81496	[Vibrio harveyi]	AAC46383.1
LlChi	[Lactococcus lactis subsp. lactis Il1403]	AAK06048.1
Pfpu7ChiC	[Paenibacillus sp. FPU-7]	BAM67139.1
NpChi	[Nocardiopsis prasina]	BAC45251.1
ScChiA	[Streptomyces coelicolor A3(2)]	CAB92596.1
SlChiA- D13775	[Streptomyces lividans]	BAA02918.1
ScChiJ	[Streptomyces coelicolor A3(2)]	CAB69724.1
AeroChi c- BAA09628.1	[Aeromonas sp. 10S-24]	BAA09628.1
CvioChiA	[Chromobacterium violaceum ATCC 12472]	AAQ60603.1
VcChiA	[Vibrio cholerae]	AAC72236.1
XAKChiA	[Xanthomonas sp. AK]	BAA36460.1
ScChiB	[Streptomyces coelicolor A3(2)]	CAA20216.1
SlChiB- D84193	[Streptomyces lividans]	BAA25139.1
Soli01- X71080	[Streptomyces olivaceoviridis]	CAA50398.1
AeroChi a- BAA09626.1	[Aeromonas sp. 10S-24]	BAA09626.1
CsChiE	[Chitiniphilus shinanonensis]	BAK53889.1
PeChi b	[Paenibacillus elgii]	WP_010496918.1
BcW12ChiD1- D10594	[Bacillus circulans]	BAA34114.1
PeChi a	[Paenibacillus elgii]	WP_010500590.1
BtChi d	[Bacillus thuringiensis serovar kurstaki str. HD73]	AGE79587.1
BcerExoChi	[Bacillus cereus ATCC 14579]	AAP10651.1
BcerChiCH	[Bacillus cereus]	AAP47142.1
BcerNCTU2	[Bacillus cereus]	ACY39278.1

Table S6: List of abbreviations and enzyme sources with GenBank accession numbers.

Supplementary Fig. 1

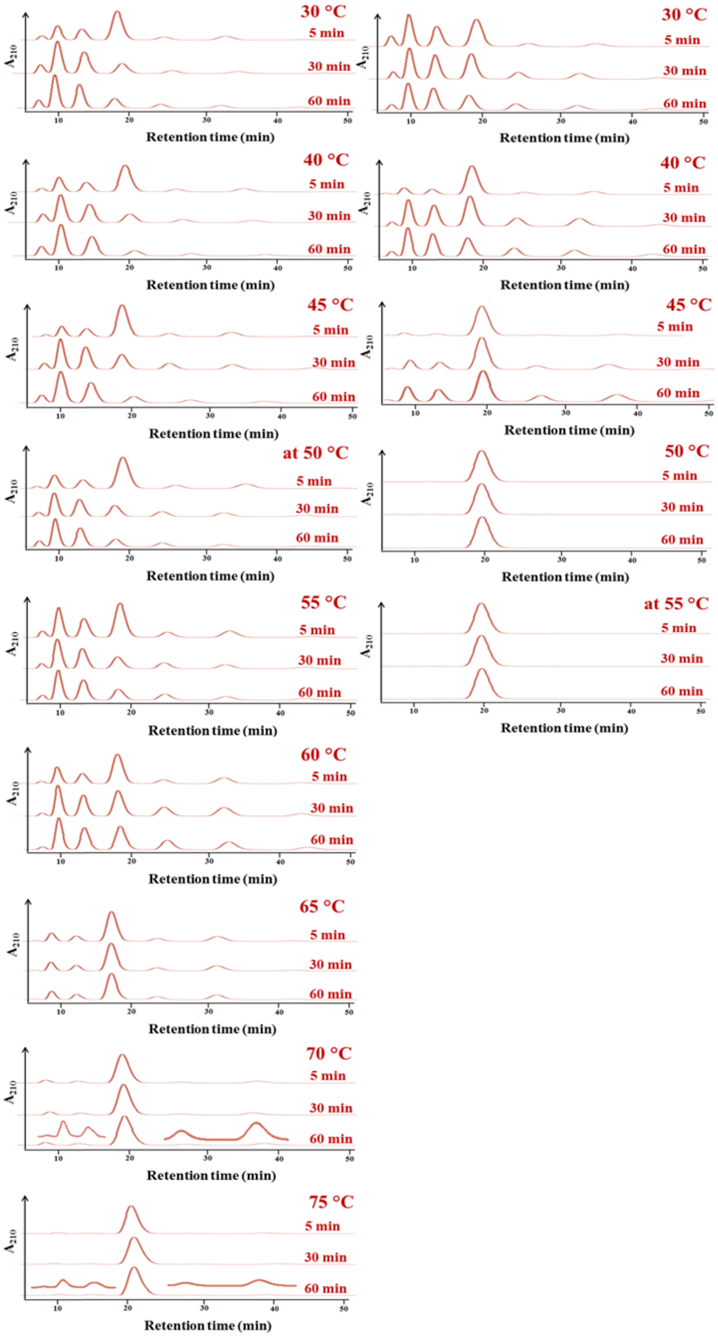

Fig. S1: **Effect of temperature on *Sp*ChiD activity as determined by HPLC.** In two different conditions, the ligand and/or enzyme were pre-incubated for 20 min at different temperatures and the reaction progress was monitored by HPLC. Figures on the left represent substrate pre-incubation and those on the right represent enzyme pre-incubation. Enzyme pre-incubation showed a decline in activity at 45°C and a complete loss at 50°C, whereas substrate pre-incubation showed ligand-induced thermal stability up to 60°C and a decrease in the activity from 65°C.

Supplementary Fig. 2

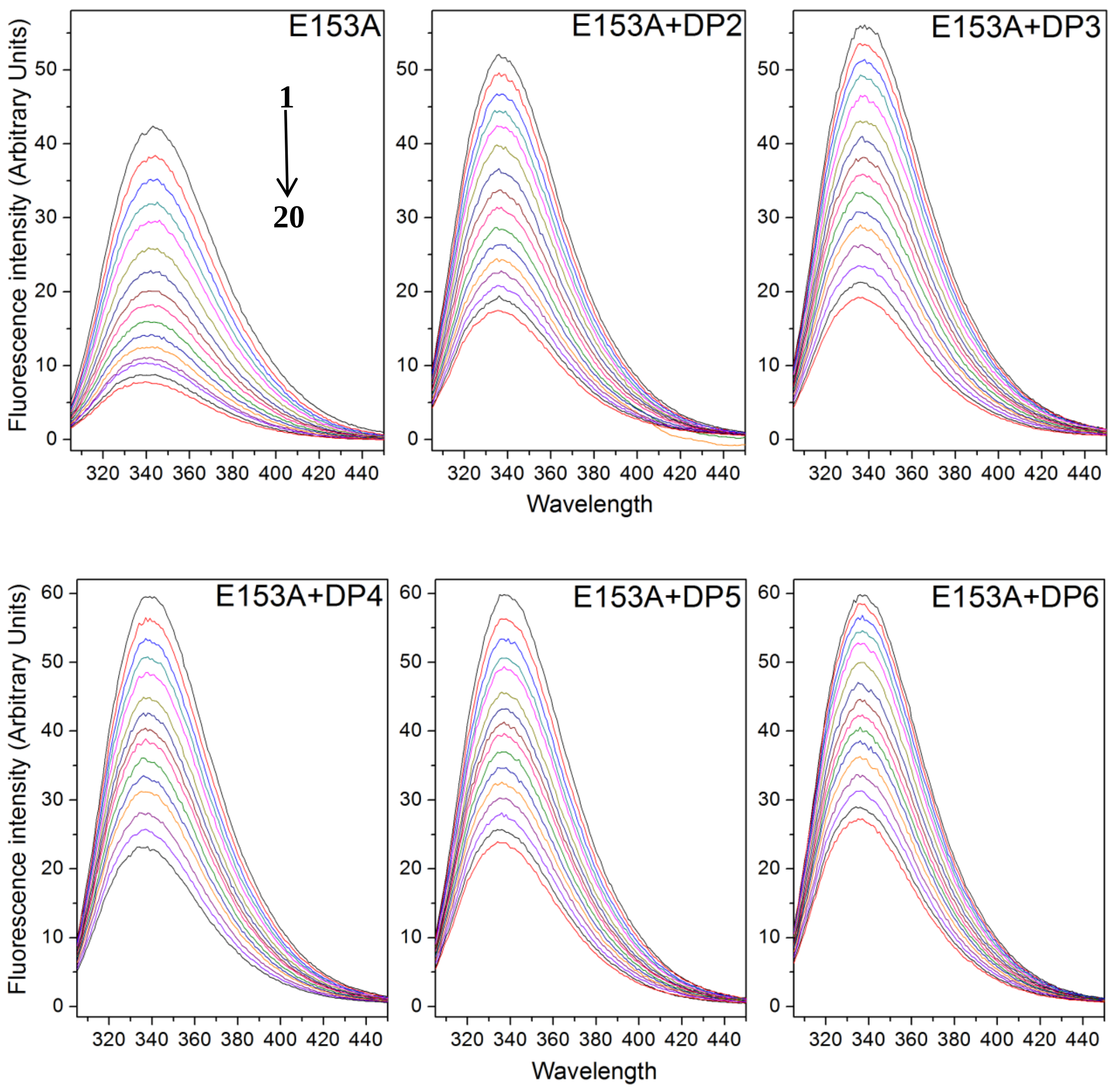

Fig. S2: **Fluorescence quenching studies with E153A.** Fluorescence emission spectra corresponding to quenching experiments carried out in the absence and in the presence of CHOS with DP2–6. In the representation 1→20, 1 corresponds to the spectrum recorded for protein alone and 2–20 correspond to increasing concentrations of the quencher acrylamide. All spectra were recorded under the same conditions.
